# Spatial inequality and explaining the urban-rural gap in obesity in India: Evidence from 2015–16 population-based survey

**DOI:** 10.1371/journal.pone.0279840

**Published:** 2023-01-04

**Authors:** Somdutta Barua

**Affiliations:** 1 Centre for the Study of Regional Development, Jawaharlal Nehru University, New Delhi, India; 2 National Institute of Urban Affairs (NIUA), New Delhi, India; Banaras Hindu University, INDIA

## Abstract

**Objective:**

This study assessed the spatial dimension of urban-rural disparity in obesity prevalence and identified the determinants explaining the urban-rural gap in obesity prevalence in India.

**Methods:**

Using cross-sectional survey data from the 2015–16 National Family Health Survey, the prevalence rates of obesity were calculated for aged 15–49 years. Two multiscale geographically weighted regressions were performed separately from rural and urban spaces for Indian districts to examine the spatial relationship of the outcome variable and covariates at different geographical scales. Fairlie decomposition analysis was carried out to explore the contribution of each variable in the urban-rural gap.

**Results:**

The rural-urban obesity prevalence difference has increased in a decade time for India from 13.0 to 14.6. Urban counterparts tended to have more people with excess weight. 15 states had an urban-rural prevalence ratio of 2 or higher. The MGWR model showed that varying covariates operated at different scales, i.e. global, regional and local scales, and determined the spatial heterogeneity of obesity prevalence. The only variable, i.e. age (9.49 per cent), had contributed in reducing the gap. Conversely, the socioeconomic variables, i.e. income (96.39 per cent), education (4.95 per cent), caste (4.78 per cent) and occupation (3.11 per cent), had widened the gap.

**Conclusions:**

Even though this study evidenced the rural-urban gap in obesity prevalence, it indicated the gap’s closing shortly, as it was witnessed in a few states. It is urgent to address the obesity epidemic, especially in urban India, due to its higher prevalence and prevent the further increase of prevalence in rural India, mainly because it shelters nearly 70 per cent of the Indian population.

## Introduction

The World Obesity Federation (WOF) recognises obesity as a disease that requires proactive preventive efforts [1], while the World Health Organization has described obesity as one of the most neglected public health issues at present [2]. The prevalence of obesity has increased three times from 1975 to 2016 with a global adult population of 13 per cent (15 per cent women and 11 per cent men), i.e. 650 million people have been living with obesity [3]. This increasing prevalence creates a massive threat to public health [4]. It can affect overall health, i.e., physical, mental/psychological, social and cultural, emotional and spiritual health [5]. It tends to increase the risk of other non-communicable diseases [4] and reduce disability-free life expectancy and total life expectancy [6]. The known list of associated complications resulting from excess fat are hypertension, dyslipidaemia, type 2 diabetes, coronary heart disease, gallbladder disease, stroke, sleep apnea, osteoarthritis, cancers, respiratory problems, polycystic ovarian syndrome, gallstones, pregnancy complications and depression [7] and many more. Even individuals with BMI over 25 exhibited a higher risk of contracting Covid-19 [8], with a 50 per cent increased mortality risk [9]. Further, obesity may result in low quality of life, functional impairment, loss of productivity, work absenteeism, psychosocial problems, etc.

The problem of obesity started in the United States and other high-income countries before it spread to middle- and low-income countries. Prevalence was lower in Asian countries; however, presently, the rates are rapidly rising in India and China, especially in urban areas [6]. The accelerated increase in obesity rates in the developed and developing world in the last few decades is due to the enormous changes in food patterns in conjunction with the associated factors at the global, population, and individual levels. The transition started when processed foods full of saturated fats, sugar and sodium replaced the traditional healthy diet of whole grains, lean protein, and vegetables and the increased sedentary activities due to the change in occupational structure from rural subsistence to low activity lifestyle. That explains why there are more people with excess weight compared to the past [7]. Barry Popkin [10] has asserted that the known main reason for humans becoming fatter during the last few decades are our diet mainly containing fat, sugar and high-calorie drinks; sedentary lifestyle; urbanisation; increasing stable accessibility to low-cost processed foods and also the transition from agriculture to an industrialised economy. Notable economists Thomas Philipson and Richard Posner have mentioned that the increase in obesity is the function of the mass-produced meal of the improved technology [11].

In developing countries, 115 million or more people suffer from obesity-related issues [2]. Obesity prevalence is likely to increase further as socioeconomic transitions take place before it shifts toward the socioeconomically disadvantaged group. That is because developing countries are on the verge of adopting a westernised lifestyle, characterised by increased calorie-dense food intake and decreased energy expenditure, which is more common among the affluent class [11, 12]. Fast food has become an affordable option for wealthy urban residents in nations like China and India, where economic growth has recently begun [13–16]. Given that diet and physical activity are the two main risk factors of overweight and obesity, the country’s continued economic development may increase sedentary lifestyles, higher incomes, and greater availability of processed food in urban areas [17, 18]. People living in cities are mostly a part of the cash economy, with increased technology and a sedentary job profile. They have easy access to unhealthy pre-packed processed food, with the global food structure working against the time taken to shop, prepare and cook healthy food [7].

The NCD Risk Factor Collaboration [19] research study showed that a global share of 5.3 per cent (20 million) of women and 3.7 per cent (9.8 million) of men were present with obesity in India in 2014, ranking 3rd and 5th, respectively. In three and a half decades, the percentage rose from just 0.8 per cent (1.2 million) in females and 0.4 per cent (1.3 million) in males, significantly rising from the 19th rank. Moreover, it would be challenging for developing nations like India to tackle chronic disabilities co-occurring with obesity in poor healthcare settings [7]. According to the World Obesity Federation, by 2025, India will be spending the US $13 million annually on obesity-related illnesses and its treatment [20]. India has been undergoing a demographic, epidemiological, and nutrition transition process in most parts of India since 1990 [21]. In India, around 20 per cent of the population aged 15–49 years have a BMI of 25 or higher, with 241 out of 640 districts having a higher percentage than the national average [22]. According to NFHS 2015–16, urban counterparts are more likely to have individuals with excess weight than rural residents [23]. Few studies [21, 24] suggest that obesity is much higher in urban than in rural areas in India. Even though a recent study [21] implies the reduction of the rural-urban gap, wider differences are still found in India. No prior research has been conducted on the spatial dimension of rural and urban obesity prevalence in India. Further, no study methodically explored the factors explaining the rural-urban gap in obesity prevalence. It is crucial to study the factors resulting in the higher prevalence in urban than rural counterparts, mainly because the urban population is increasing. Since there is a lack of systematic attempts, this study has attempted to study the spatial dimension of urban-rural disparity in obesity prevalence at the state and district level. Further, it has identified the determinants explaining the urban-rural gap in obesity prevalence in India at the individual level.

## Materials and methods

### Data source

The study utilised data from the National Family Health Survey (NFHS-4), a cross-sectional survey conducted in India during 2015–16, capturing data on key population indicators such as health, family welfare, and related issues at the district level. The NFHS-4 is a nationally representative large-scale sample survey including 601,509 households, 112,122 men aged 15–54 years and 699,686 women aged 15–49 years. NFHS-4 designed a stratified two-staged sample, using census 2011 as the sampling frame for selecting PSUs. In this survey, weight and height were taken for women and men aged 15–49 years and 15–54 years, respectively [22].

### Study participants

The final sample size was 738,265 respondents who had given height and weight measurements. This study included 6,38,265 women and 99,609 sampled men aged 15–49 years. Pregnant women were dropped from the present analysis.

### Dependent variable

The outcome variable of this study is the excess weight (having BMI = >25) among women and men aged 15–49 years. BMI was calculated from the height and weight measurements, using NFHS- 4 data in Stata 14.0 for women and men aged 15–49 years. The World Health Organization, the International Obesity Task Force and the International Association for the Study of Obesity recommended a BMI of 25 to be the cut-off for obesity for Asian populations since a BMI of 30 tended to underestimate the body fat level in Asians [7]. Hence, in this study, following the same guidelines, individuals were categorised as having excess weight when their BMI was 25 or higher. Height and weight are still highly prevalent in epidemiological studies because of their inexpensiveness [6].

### Independent variables

Several socio-demographic variables and calorie-dense foods are included as confounding factors that tend to have a potential impact on the prevalence of excess weight. The independent covariates that could confound obesity prevalence were several non-clinical such as age, sex, marital status, education, income (wealth index), caste, religion, occupation, milk, pulse, vegetable, fruit, egg, fish, meat, aerated drinks, and fried foods, were initially considered as per data availability, and online literature. The Multicollinearity Condition Number (MCN) provided by the OLS output served as the foundation for the final list of covariates. However, while using the district-level data where the dependent variable was district-level obesity prevalence, all of the previously listed variables resulted in an MCN of greater than 30. As a result, 7 variables were ultimately chosen for the final main analyses in this study to maintain uniformity. The independent variables of this study include age, educational level, income, caste, occupation, daily consumption of aerated drinks and fried foods.

### Method of analysis

The ‘prevalence of obesity’ was the ratio of the number of persons who had a BMI of 25 or higher at the time of the survey to the total number of persons who had given measurements of height and weight. For the estimation of the percentage distribution, sample weight was applied. In the national report of NFHS-4 and NFHS-3, sample weight has been explained in detail [22, 25]. The maps were prepared using ArcGIS 10.8. Further, two multiscale geographically weighted regressions were performed separately from rural and urban spaces for Indian districts to examine the spatial relationship of the outcome variable and covariates at different geographical scales. All of the included variables were standardised within the MGWR software. The MGWR estimates a separate regression for each observation, with a different weighting. Observations from nearby locations have a stronger influence (Tobler 1970). Each observation is assigned a weight based on a distance decay function between the county centroids [26]. MGWR allows the assessment of the multiscale processes that employ varying bandwidths for every covariate [27]. The mathematical form of the MGWR model can be expressed as

$$yi=\sum_{j=0}^{m}\beta_{bwj}(u_{i},v_{i})+\varepsilon_{i},$$

where, *bmj* is the bandwidth used for calibration of the *jth* conditional relationship.

MGWR model is considered more robust as all the parameters are processed at a varying and more flexible scale, hence more reliable. In the MGWR model, the adaptive bandwidth technique was employed. The optimal bandwidths for intercept and covariates were determined using the AICc values. The bandwidths of the MGWR aid in understanding at which geographic scale each process of parameters operates. MGWR employed different kernel bandwidth for each covariate to capture multi-scale processes [28]. The study used a first-order queen contiguity matrix for building a spatial weight matrix for all the districts. This study’s possible largest bandwidth is 628 for rural and 638 for urban. That is the total number of the district in the study area for rural and urban spaces. These analyses were performed with the help of Stata 14.0, ArcGIS 10.8, Geoda and MGWR 2.2 softwares. Finally, fairlie decomposition analysis has been used to examine the contribution of each variable in the urban-rural gap. Blinder-Oaxaca decomposition is one of the most common techniques used for identifying and quantifying the separate contribution of group differences in measurable characteristics. As per the Standard Blinder-Oaxaca decomposition of the urban-rural gap in the average value of the dependent variable, Y, can be expressed as

$${\bar{Y}\;}^{U}-\;{\bar{Y}\;}^{R}=\left[({{\bar{X}\;}^{U}}^{\;}-{\bar{X}\;}^{R}){\hat{\beta }}^{U}\right]+\left[\;{\bar{X}\;}^{R}({\hat{\beta }}^{U}-{\hat{\beta }}^{R})\right]$$

where ${\bar{X}\;}^{j}$ is a row vector of average values of the independent variables and ${\hat{\beta }}^{j}$ is a vector of coefficients estimates for place of residence ***j***.

However, a problem occurs if the outcome is binary, such as obesity (yes or no), and the coefficients are from a logit or a probit model. Hence, a relatively simpler method of decomposition, i.e. fairlie decomposition, was introduced by Fairlie, which could use the estimates from a logit or a probit model [29].

Thus, an extension of this decomposition for a non-linear equation, $Y=F(X\hat{\beta })$, can be written as

$${\bar{Y}\;}^{U}-\;{\bar{Y}\;}^{R}=\left[\sum_{i=1}^{N^{U}}\frac{F\left(X_{i}^{U}\right.\left.{\hat{\beta }}^{U}\right)}{N^{U}}\right.-\left.\sum_{i=1}^{N^{R}}\frac{F\left(X_{i}^{R}\right.\left.{\hat{\beta }}^{U}\right)}{N^{R}}\right]+\left[\sum_{i=1}^{N^{R}}\frac{F\left(X_{i}^{R}\right.\left.{\hat{\beta }}^{U}\right)}{N^{R}}-\left.\sum_{i=1}^{N^{R}}\frac{F\left(X_{i}^{R}\right.\left.{\hat{\beta }}^{R}\right)}{N^{R}}\right]\right.$$

An equally valid expression for the decomposition is:

$${\bar{Y}\;}^{U}-\;{\bar{Y}\;}^{R}=\left[\sum_{i=1}^{N^{U}}\frac{F\left(X_{i}^{U}\right.\left.{\hat{\beta }}^{R}\right)}{N^{U}}\right.-\left.\sum_{i=1}^{N^{R}}\frac{F\left(X_{i}^{R}\right.\left.{\hat{\beta }}^{R}\right)}{N^{R}}\right]+\left[\sum_{i=1}^{N^{U}}\frac{F\left(X_{i}^{U}\right.\left.{\hat{\beta }}^{U}\right)}{N^{U}}-\left.\sum_{i=1}^{N^{U}}\frac{F\left(X_{i}^{U}\right.\left.{\hat{\beta }}^{R}\right)}{N^{U}}\right]\right.$$

Here, to calculate the decomposition, define ${\bar{Y}\;}^{j}$ as the average probability of the binary outcome of the interest group ***j*** and ***F*** as the cumulative distribution function from the logistic distribution. ‘U’ represents urban residence, ‘R’ represents rural residence, and ‘N’ represents the sample size. The first terms in the second and third equations estimate the contribution of urban-rural difference in the entire set of independent variables to the urban-rural gap in obesity prevalence. To get the total contribution, the calculation needs to be done for two sets of predicted probabilities by urban-rural and take the difference between the average values of the two [29, 30]. This analysis was performed on Stata 14.0.

Therefore, this technique is useful when it is inappropriate to model the dependent variable as the linear function of the independent variables. Hence, the fairlie decomposition is performed (unweighted no = 2,00,77) to explain the urban-rural gap in the prevalence of obesity to analyse the relative contribution of varying background characteristics.

## Results

### Inter-state variation of the urban-rural difference in obesity prevalence

**Fig 1:**
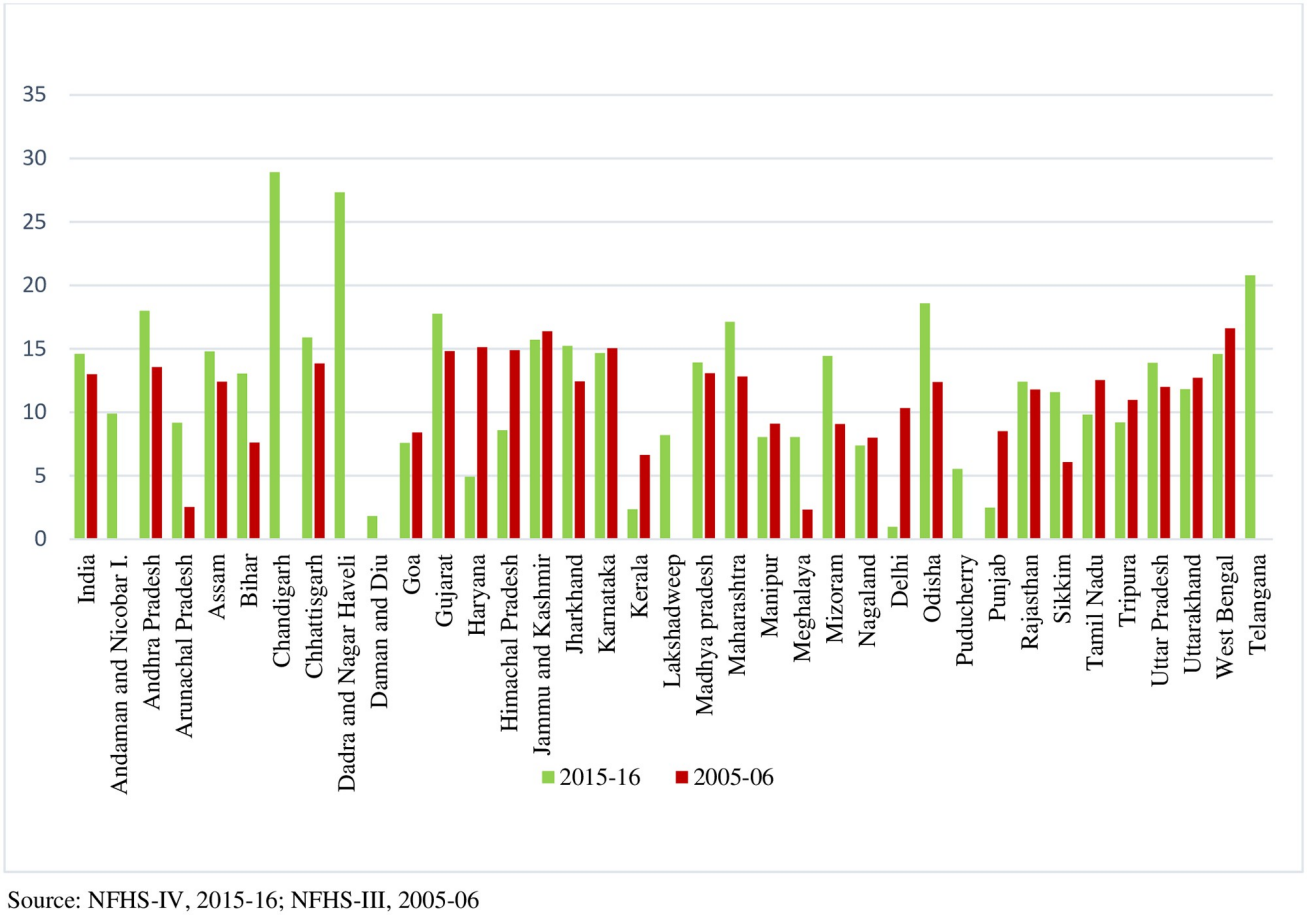
Inter-state variation of urban-rural prevalence difference. Source NFHS-IV, 2015–16; NFHS-III, 2005–06.

Fig 1 presents the state-level urban-rural prevalence difference in obesity for aged 15–49 years in 2005–06 and 2015–16. The urban-rural obesity prevalence difference has increased in a decade time for India from 13.0 to 14.6. The urban-rural prevalence difference has increased for 19 states positively, whereas it has decreased for 10 states from 2005–06 to 2015–16. The highest increase in the urban-rural prevalence difference in obesity is witnessed in Arunachal Pradesh from 2005–06 to 2015–16. On the other hand, in 2015–16, the highest urban-rural prevalence difference can be found in Chandigarh and the lowest in Delhi.

**Fig 2:**
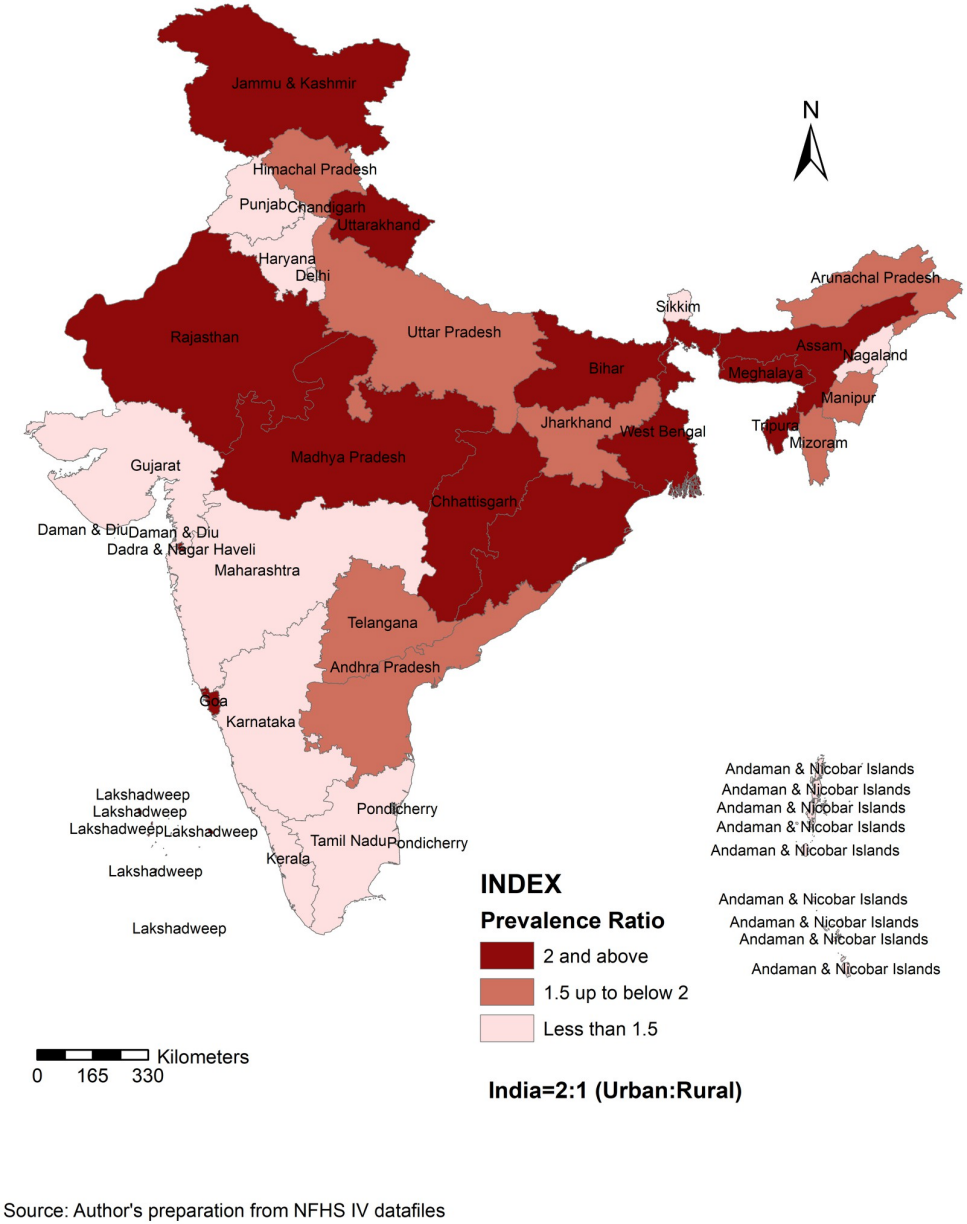
Inter-state variation of urban-rural prevalence ratio, 2015–16. Source: Author’s preparation from NFHS IV datafiles.

Fig 2 presents the urban-rural obesity prevalence ratio. 15 states have a prevalence ratio of 2 or higher, whereas 13 states have a prevalence ratio of 1.5 or lower. Dadra & Nagar Haveli has the highest prevalence ratio, and Nagaland has the lowest.

### Estimation of MGWR

The global regression models signify a moderate and weak association between the covariates and obesity prevalence in rural and urban spaces. The R^2^ in the global regression model indicated that the covariates could explain 54.2 per cent and 24.8 per cent of the variance in the obesity prevalence in rural and urban localities. On the other hand, the results of the MGWR model indicated an improved R^2^ of 0.867 and 0.633 for rural and urban counterparts, respectively, since it had considered spatial heterogeneity in the process.

In the MGWR model, the calibration gives a matrix of optimal bandwidth, which helps to understand at which geographic scale each parameter processes: global, regional or local. The factor-specific optimal bandwidth with minimum AICc values are 49, 43, 248, 43, 627, 404, 281, and 43 for higher age group (40–40 years), higher education, higher income, general social group, agricultural occupation, aerated drinks, fried foods, and the intercept, respectively in the rural spaces in the districts of India. While for urban spaces, the values are 163, 377, 637, 58, 637, 261, 403, and 43 for the higher age group (40–40 years), higher education, higher income, general social group, agricultural occupation, aerated drinks, fried foods, and intercept, respectively. To explain further, the proportion of the higher (40–49 years) age group population (rural BW = 49 vs urban BW = 163) is processed at a local scale, and the proportion of the population with higher education (rural BW = 43 vs urban BW = 377) is processed at a local scale for rural and regional scale for the urban counterpart. On the other hand, the proportion of the population with higher income (rural BW = 248 vs urban BW = 637) is processed at a regional scale for rural and at a global scale for urban India. Furthermore, the proportion of the general social group (caste) population (rural BW = 43 vs urban BW = 58) and the proportion of the population with agricultural occupation (rural BW = 627 vs urban BW = 637) are processed at a local scale since the bandwidth is similar to nearly one-third of the districts of the study area and at a global scale because the bandwidth is similar to the entire study area, respectively for both the place of residence. Besides, the proportion of the population having daily consumption of aerated drinks and fried foods are processed at a regional scale for rural and urban areas.

#### Rural

**Fig 3:**
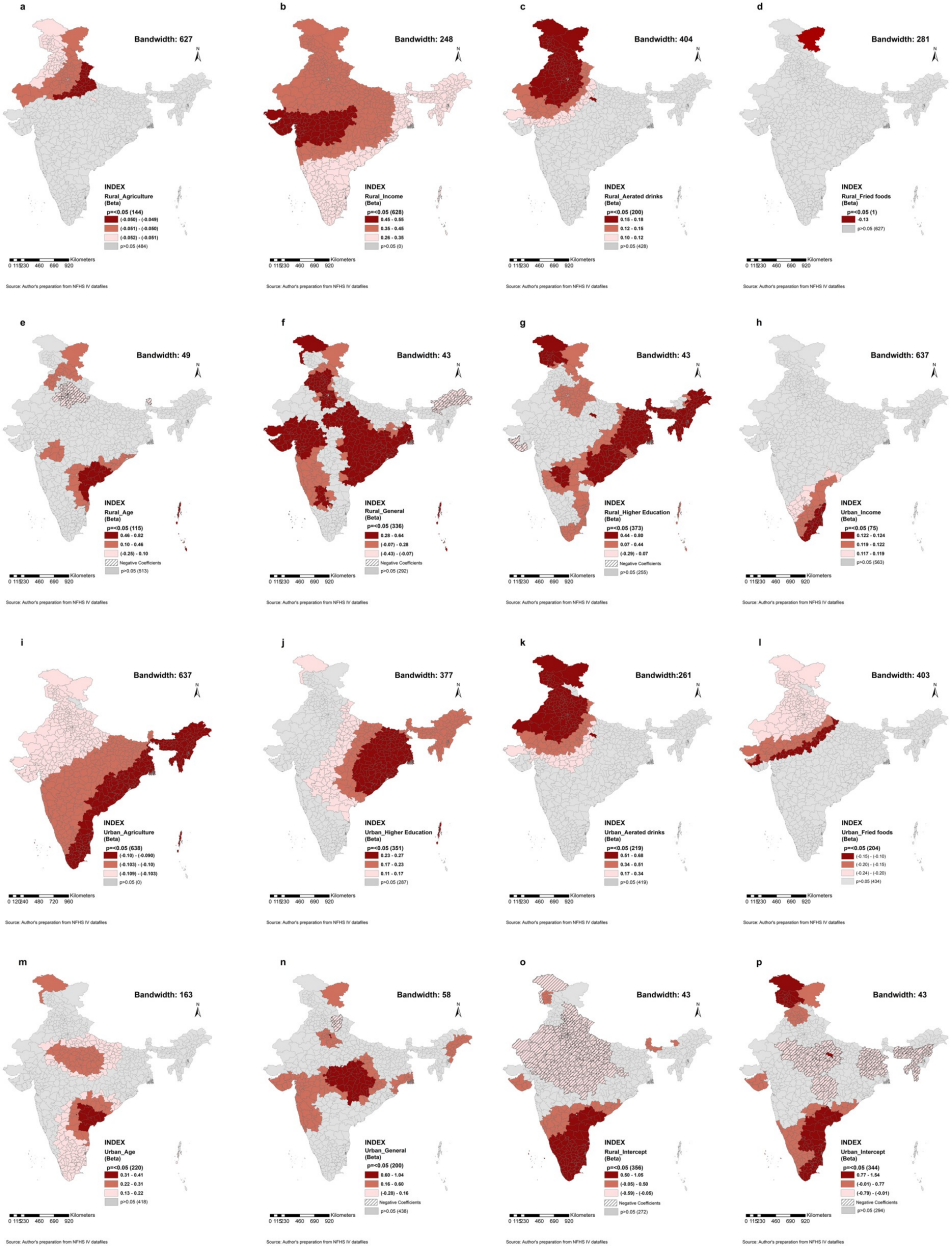
a-g. Spatial distribution of MGWR local coefficients (rural) at district level (significant areas only); the class of the local coefficient is classified by equal intervals. h-n. Spatial distribution of MGWR local coefficients (urban) at district level (significant areas only); the class of the local coefficient is classified by equal intervals. o and p. Spatial distribution of MGWR local coefficients at district level (significant areas only); the class of the local coefficient is classified by equal intervals. Source: Author’s preparation from NFHS IV datafiles.

Firstly, the effect of agricultural occupation (BW 627 nearest neighbours) on obesity prevalence is the only factor in the rural area that reflects a global scale dimension. The negative relationship between agricultural occupation and obesity prevalence significantly manifested in the districts of north India with almost no variation in the local coefficient (Fig 3a). Secondly, higher income (BW 248 nearest neighbours), daily consumption of aerated drinks (BW 404 nearest neighbours) and fried foods (BW 281 nearest neighbours) affect obesity at a regional scale. The relationship between higher income and obesity prevalence significantly manifested in all the districts of India (Fig 3b). The figure also shows that the local parameter estimates of the ‘highest wealth index’ is positive across Indian districts with little variation in the local coefficient. On the other hand, a positive relationship between aerated drinks and obesity prevalence significantly manifested in north-western India with little variation in the local coefficient (Fig 3c). Furthermore, a negative relationship between fried foods and obesity prevalence significantly manifested in only one district of India (Fig 3d). Thirdly, at the local scale, (Fig 3e) people with higher age (BW = 49), (Fig 3f) people in the general social group (BW = 43), and (Fig 3g) people with higher education (BW = 43) have positive and negative associations with obesity prevalence significantly manifested scattering in several parts of India.

#### Urban

At a global level, the population with a higher income (BW 637 nearest neighbours) is positively associated with obesity. It is significantly manifesting in the southern part of India (Fig 3h). In contrast, agricultural occupation (BW 637 nearest neighbours) is significantly manifesting negatively almost in the whole of India with almost no variation in the local coefficient (Fig 3i). At a regional scale, higher educational level (BW 377 nearest neighbours) and daily consumption of aerated drinks (BW 261 nearest neighbours) indicate a significant positive association with obesity prevalence majorly manifesting in eastern India (Fig 3j) and north-western India (Fig 3k), respectively. While the relationship between fried foods (BW 403 nearest neighbours) and obesity prevalence has a significant negative association in north and north-west India (Fig 3l) at a regional scale with little variation in the local coefficient. Reflecting on a local scale dimension, the relationship between the higher age group (BW 163 nearest neighbours) and obesity prevalence manifests a significantly positive impact, while the relationship between the general (social group) population (BW 58 nearest neighbours) and obesity prevalence manifest a significant positive and negative association that can be found in pockets in various parts of India (Fig 3m and 3n).

### Description of study participants

Table 1 presents the description of the study participants. Prevalence was higher among higher age groups, higher educational levels, and higher income groups, general social group, people of professional/technical/managerial occupation, people with daily consumption of aerated drinks and fried foods. Prevalence was found to be high in urban than rural areas for all covariates. Variation of more than 20 per cent was found for the higher age group. Differences in urban-rural prevalence were also high for individuals with primary education and OBC social group, whereas the difference in prevalence was low for the lower age group and poorest income group.

**Table 1:** Socio-demographic characteristics of study participants (n = 738265).

Background Variables	Total	BMI≥25 (%)	BMI≥25 (%) in Rural	BMI≥25 (%) in Urban
**Age**				
<20 Years	1,36,486	4.27	2.97	7.27
20–29 Years	2,33,064	14.1	10.67	20.7
30–39 Years	2,02,052	26.99	20.46	39.14
40–49 Years	1,66,663	32.78	24.16	48.62
**Education**				
No education	1,87,416	16.37	13.19	29.67
Primary	98,521	20.74	16.26	33.00
Secondary	3,65,462	20.44	15.04	29.80
Higher	86,866	25.91	18.14	31.23
**Income (Wealth Index)**				
Poorest	1,38,029	5.69	5.52	8.82
Poorer	1,57,595	11.09	10.56	15.29
Middle	1,56,712	18.37	17.07	22.24
Richer	1,47,325	27.49	24.94	30.12
Richest	1,38,604	35.42	31.05	37.04
**Social Group**				
General	1,53,590	26.37	20.11	34.15
OBC	2,85,712	20.46	15.23	30.44
SC	1,33,379	16.69	13.17	25.54
ST	1,34,900	9.89	7.75	21.8
**Occupation**				
Not employed	1,01,162	20.02	14.8	28.48
Professional/technical/managerial	8,607	33.55	26.37	38.57
Clerical	2,196	26.34	20.53	31.25
Sales	10,363	30.7	24.97	34.92
Agriculture	45,739	13.67	12.91	23.44
Services	10,511	26.11	20.63	31.06
Skilled and unskilled manual	32,176	19.54	14.76	25.97
**Aerated Drinks**				
No	6,57,186	20	14.85	30.42
Yes	81,079	20.77	15.48	30.66
**Fried foods**				
No	6,96,276	20.13	14.88	30.52
Yes	41,989	20.94	15.31	29.25
**Locality**				
Urban	2,16,424	30.44		
Rural	5,21,841	14.9	N/A	N/A

**Source**: Computed from NFHS IV data files

### Decomposition of change in the prevalence of obesity between the rural and the urban residence

Table 2 presents the result of the fairlie decomposition model. The prevalence of obesity in the urban locality is 30.44 per cent, whereas it is 14.9 per cent in the rural area; thus, a difference of 15.54 per cent is observed. Fairlie decomposition technique, an extension of the Blinder-Oaxaca decomposition technique is useful for binary models. Therefore, the fairlie decomposition technique is relevant in studying the relative contribution of varying background characteristics to the urban-rural gap in obesity prevalence.

**Table 2:** Decomposition of rural-urban gap in obesity prevalence in India, NFHS, 2015–16.

Background Characteristics	Contribution
**Demographic Variables**	
Age	-8.83***
**Socioeconomic Variables**	
Education	4.60***
Income (Wealth Index)	89.61***
Caste	4.44***
Occupation	2.89***
**Dietary Variables**	
Aerated Drinks	0.18
Fried Foods	0.06

Explained gap: 92.94 (72.31%)

Total gap: 128.53

Number of observations (n): 2,00,77

Note:

*** p<0.01,

** p<0.05 &

* p<0.10;

Coefficients are multiplied by 1000

**Source**: Computed from NFHS IV data files

**Fig 4:**
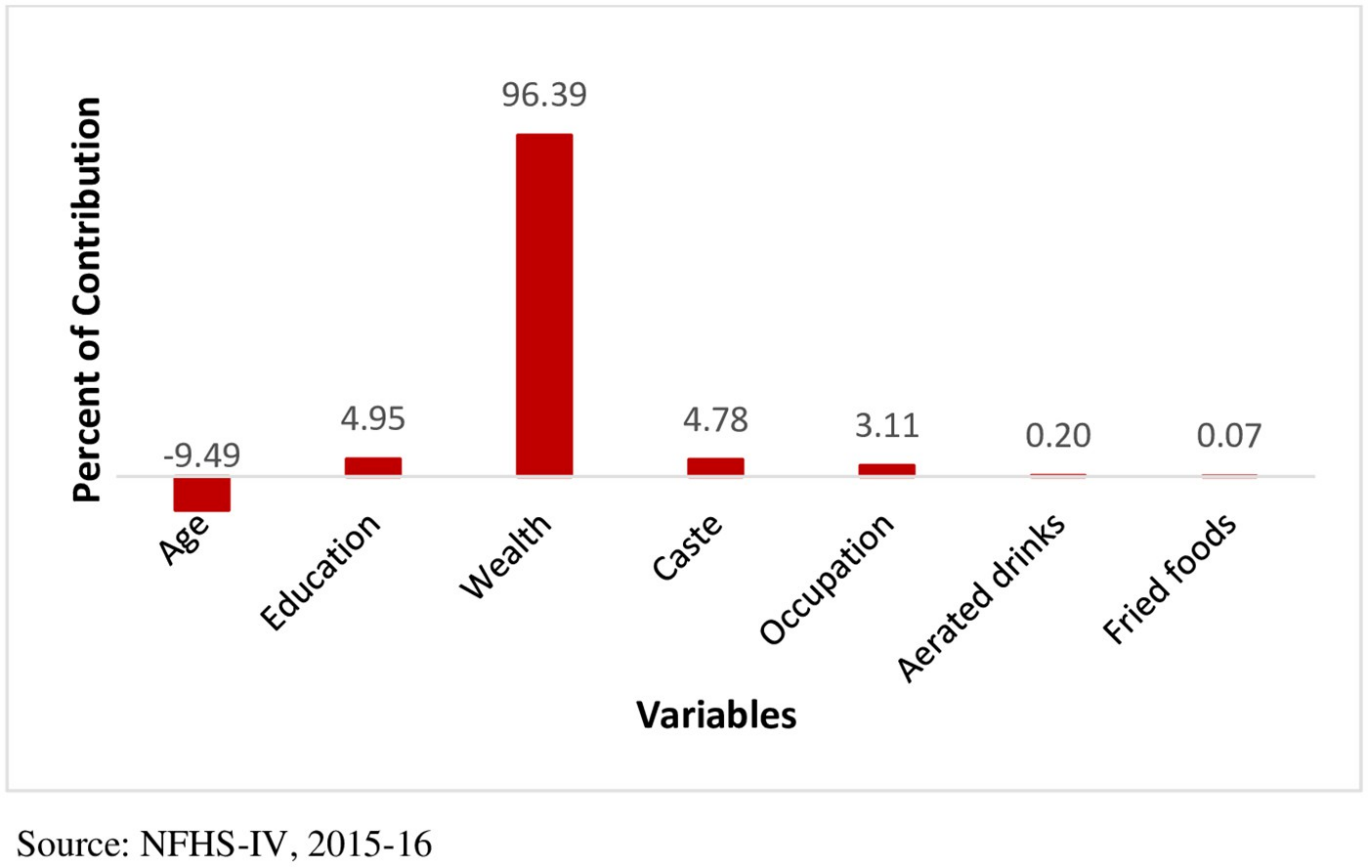
Percentage contribution of each variable to the rural-urban gap in obesity prevalence in India, 2015–16. Source: NFHS-1V, 2015–16.

Table 2 presents the comprehensive results of a decomposition analysis of the urban-rural gap in the prevalence of obesity by the independent variables. The coefficients are multiplied by 1000. The positive contribution of the background variables implies that a particular variable (e.g. income) widens the gap in obesity prevalence between urban and rural localities. In contrast, the negative contribution of a variable (e.g. age) suggests that the variable contributes in reducing the gap. The results also depict that 72.31 per cent of the difference in obesity prevalence between the urban and rural regions is explained by the variations in the distribution of independent variables. However, this study could not include all other variables, such as physical activity, waist circumference, parental history of obesity, etc. The information regarding these parameters was not available in the data set. On the other hand, some covariates such as sex, marital status, religion, cancer, asthma, thyroid, heart disease, daily consumption of alcohol, milk, pulse, vegetable, fruit, egg, fish, and meat were excluded since their exclusion led to a Multicollinearity Condition Number >30. Therefore, it could be the possible reason for the unexplained gap in the rural-urban disparity. The demographic variable, i.e. age, is the only factor contributing 9.49 per cent in reducing the gap (Fig 4). In contrast, the socioeconomic variables have contributed the maximum in widening the gap between the urban and rural localities. Income (wealth index) contributes the highest (96.39%) in increasing the urban-rural gap. Education, caste, and occupation have also been observed to widen the gap considerably.

## Discussion and conclusions

This study aimed to identify states in India with high urban-rural gap, study the spatial dimension of urban-rural disparity in obesity prevalence, and explore the factors explaining the urban-rural gap in obesity prevalence to facilitate needed intervention. This information can be vital to understanding which state needs immediate intervention and requires policy briefing. A graph and map have been created detailing the obesity prevalence difference and prevalence ratio across Indian states. Further, using the MGWR model, the determinants of obesity in rural and urban spaces were studied at the district level, while the fairlie decomposition was performed to explain the urban-rural gap in the prevalence of obesity to analyse the relative contribution of varying background characteristics. The study’s strength is that it is based on fairly recent large-scale data where height and weight measurements are taken for every participant. The important findings are:

Firstly, the urban-rural obesity prevalence difference has increased in a decade time for India from 13.0 to 14.6 and in many states, with urban people tending to have more people with excess weight in 2015–16 than in 2005–06. That means in 2005–06, there were 13 more people with excess weight in urban localities per 100 compared to the rural counterparts, whereas it increased to around 15 in 2015–16 in India. The 19 states, e.g. Arunachal Pradesh (highest) and Rajasthan (least), witnessed an increase in the urban-rural prevalence difference, which means the urban-rural gap increased in these states from 2005–06 to 2015–16. On the contrary, for another 10 states, the urban-rural prevalence difference has decreased from 2005–06 to 2015–16, which perhaps indicates that the rural prevalence is rising, and hence the urban-rural prevalence gap has closed for these states, e.g. Delhi. The highest decline from 2005–06 to 2015–16 was witnessed in Haryana and the least in Karnataka. Furthermore, the prevalence in the urban was much higher than in the rural counterparts, with a 2:1 urban-rural prevalence ratio found in India in 2015–16. An urban-rural obesity prevalence ratio map was created detailing the urban-rural gap. A similar prevalence was only found in Nagaland since the ratio was 1:1, whereas the ratio was much higher in Dadra and Nagar Haveli.

The higher prevalence in the urban area is comparable with two national-level studies [21, 31], one local-level study [24] in India and one study in a developing country [18]. Chandigarh had the highest prevalence difference among all other Indian states/UTs in 2015–16, perhaps due to its highly urbanised nature and the lifestyle changes that accompanied the process of urbanisation. A prior study [32] documented that a transition in dietary and physical activity patterns from rural to urban India could be a significant cause of the obesity epidemic in the country. On the other hand, perhaps Delhi has been witnessing a faster and higher rise in rural prevalence of obesity than the urban; hence the gap tends to close between the rural and urban. It is plausible that the rising standard of living in Delhi, the capital city of India and the accompanying lifestyle changes have been leading to the increasing prevalence in the rural counterparts as well. Similarly, Haryana sharing its border with the capital city shows a similar pattern. Conversely, Arunachal Pradesh located in the northeastern part of India, has observed an increase in the urban-rural gap from 2005–06 to 2015–16. That probably indicates that the urban-rural gap is increasing in India’s economically less developed states.

Secondly, the multi-bandwidth assisted in producing a more accurate model that explored the determinants of obesity prevalence across Indian districts. The MGWR model showed how varying factors operated at different scales in determining the spatial heterogeneity of obesity prevalence. Hence, it is evident that spatial disparity exists in India. The local parameter estimates of these variables can be readily compared to one another because all of the study’s variables have been standardised [33]. At a regional and a global scale, the districts with shades of red illustrate that individuals with higher income are at higher risk of having excess weight in rural and urban spaces, respectively. The finding that wealth is positively associated with obesity is consistent with previous studies in India [21, 31] and developing countries [18]. Suitable global measures should be targeted for the cluster identified in the south of India for urban areas. On the other hand, for rural areas, it is necessary to target the region with positive coefficients to reduce the prevalence rate. In the present study, higher age populations are positively and negatively associated with obesity in rural India and only positively in urban India. Prior studies [21, 34, 35] have also found that higher age is positively associated with excess weight. The significance is very localised; hence local interventions are necessary to prevent and reduce excess weight, especially in the districts with positive coefficients. Similarly, the general population are positively and negatively associated with obesity. Local interventions are needed to prevent and reduce excess weight among the general population in urban and rural India, especially in the districts with positive coefficients. The general population tend to be an economically privileged group. Having said so, they are more likely to have excess weight because wealth is positively linked with obesity in developing countries [18] like India [21, 31]. The effect of agricultural occupation on the prevalence of obesity witnessed a global scale dimension. The negative relationship manifested significantly in the north-western part of India for the rural areas and illustrated the global nature almost across entire India for the urban counterparts. A study stated that workers in certain occupations were less likely to have excess weight [36], and the potential for occupational physical activity on the total caloric expenditure could have a much greater impact [37]. Hence the negative association is perhaps due to higher involvement in physical activity and energy expenditure with agricultural occupation. Furthermore, for the maximum number of districts, individuals with higher education are at higher risk of having excess weight, as also can be found in the literature [18, 31]. Since the higher educational level is working at a local and regional scale, local interventions are necessary to prevent and reduce obesity, especially in the districts with positive coefficients in rural areas. In contrast, interventions at a regional scale are needed to target the urban clusters identified in India with shades of red. According to the global obesity observatory, obesity prevalence rises with increasing education in lower-income countries, while the opposite is true for higher-income countries. It is because of the complicated relationship between socioeconomic status, income and education, and other determinants like an urban environment, health care setting, affordability, and accessibility to healthy foods [38]. At a regional scale, the daily consumers of aerated drinks are at risk of obesity in urban and rural areas. Suitable interventions should be targeted for the shades of red clusters identified in north and north-western India to prevent and reduce excess weight. Prior studies in India also stated that the prevalence of excess weight tended to be higher among consumers of aerated drinks [39, 40]. At a regional scale, the population who are the daily consumers of fried food have a negative association with obesity in both urban and rural areas. However, it is only significant in a single district in non-urban areas and the north-western region of India for its urban counterparts. Moreover, the intercept map (3o & 3p) demonstrated that even when no variables were included, parts of India, such as spreading over north and south, showed a risk of obesity. The local intercept could aid in developing microscale intervention policies. It is evident from the results that obesity prevalence is spatially heterogeneous, determined by the factors that vary at global, regional and local scales, and requires scalable interventions. Identification of the districts with higher prevalence is essential to prevent excess weight at a localised scale. Regional planning could take a cue from the results to prevent and reduce excess weight.

Finally, the results given by the fairlie decomposition analysis reveal that the higher prevalence of obesity in the urban region denotes the unequal distribution of various background variables across the living residences. The demographic factor, age, contributed in reducing the urban-rural gap among all other variables. The simplest way to explain it is that people age irrespective of the locality of residence; hence it tends to reduce the rural and urban gap. On the contrary, among the socioeconomic characteristics, income (wealth index), education, caste, and occupation have contributed highly and significantly in widening the urban-rural gap. The wealth index is the most significant factor in increasing the rural-urban gap in obesity prevalence. Two prior studies based in developing nations [18, 35], like India, witnessed a higher prevalence of obesity among people with higher socioeconomic status, a scenario opposite to that of a developed nation. Balasubramanian et al. [41] also mentioned the rural-urban gap in income was two times higher in urban India than in rural India. Therefore, as persons living in the urban locality tend to have a better income, higher education, and belong to the privileged caste than their rural counterparts, it has increased the rural-urban gap in obesity prevalence. Occupation is also an important factor increasing the rural-urban disparity in obesity prevalence, plausibly because of the varying occupational structure of India’s urban and rural residents.

The prevalence of obesity is likely to escalate in India in the absence of tailored intervention. It is urgent to address the obesity epidemic, especially in urban India, due to its higher prevalence and prevent the further increase of prevalence in rural India, especially because it shelters nearly 70 per cent of the Indian population. Even though this study evidenced the rural-urban gap, it indicated the closing of gap in the near future, as witnessed for a few states. Raising awareness and health education is required, not only to prevent obesity but it would also help to keep check its associated complications and diseases to keep the medical burden to minimum. Policymakers and other stakeholders should consider the identified districts in this study to develop effective policies to reduce the prevalence of obesity. Further, knowledge of the factors playing its role in the living locality (rural and urban) that increase the risk of obesity can help develop well-formulated targeted policies. An important limitation of this study was that since the inclusion of any of these variables, sex, marital status, religion, cancer, asthma, thyroid, heart disease, daily consumption of alcohol, milk, pulse, vegetable, fruit, egg, fish, and meat led to a Multicollinearity Condition Number >30, these variables were dropped from the analysis. Furthermore, some factors that could have been important determinants of obesity could not be included in the study due to the unavailability of data: physical activity, waist circumference, parental history of obesity, etc. However, this study adds to the current literature on obesity based on a large nationally representative sample, and the study findings could help form evidence-based interventions.
